# HFA-BDP Metered-Dose Inhaler Exhaled Through the Nose Improves Eosinophilic Chronic Rhinosinusitis With Bronchial Asthma: A Blinded, Placebo-Controlled Study

**DOI:** 10.3389/fimmu.2018.02192

**Published:** 2018-09-25

**Authors:** Yoshiki Kobayashi, Hirotaka Yasuba, Mikiya Asako, Takahisa Yamamoto, Hiroshi Takano, Koichi Tomoda, Akira Kanda, Hiroshi Iwai

**Affiliations:** ^1^Airway Disease Section, Department of Otorhinolaryngology, Kansai Medical University, Osaka, Japan; ^2^Allergy Center, Kansai Medical University Hospital, Osaka, Japan; ^3^Department of Airway Medicine, Mitsubishi Kyoto Hospital, Kyoto, Japan; ^4^Department of Mechanical Engineering, National Institute of Technology Gifu College, Motosu, Japan; ^5^Bio-Microfluidic Science Research Centerm, Doshisha University, Kyoto, Japan

**Keywords:** airway medicine, asthma, eosinophilic chronic rhinosinusitis, exhalation through the nose, inhaled corticosteroid, united airway

## Abstract

**Background:** Eosinophilic chronic rhinosinusitis (ECRS) is a subtype of chronic rhinosinusitis with nasal polyps in Japanese. ECRS highly associated with asthma is a refractory eosinophilic airway inflammation and requires comprehensive care as part of the united airway concept. We recently reported a series of ECRS patients with asthma treated with fine-particle inhaled corticosteroid (ICS) exhalation through the nose (ETN).

**Objective:** To evaluate fine-particle ICS ETN treatment as a potential therapeutic option in ECRS with asthma.

**Methods:** Twenty-three patients with severe ECRS under refractory to intranasal corticosteroid treatment were randomized in a double-blind fashion to receive either HFA-134a-beclomethasone dipropionate (HFA-BDP) metered-dose inhaler (MDI) ETN (*n* = 11) or placebo MDI ETN (*n* = 12) for 4 weeks. Changes in nasal polyp score, computed tomographic (CT) score, smell test, and quality of life (QOL) score from baseline were assessed. Fractionated exhaled nitric oxide (FENO) was measured as a marker of eosinophilic airway inflammation. Response to corticosteroids was evaluated before and after treatment. Additionally, deposition of fine-particles was visualized using a particle deposition model. To examine the role of eosinophils on airway inflammation, BEAS-2B human bronchial epithelial cells were co-incubated with purified eosinophils to determine corticosteroid sensitivity.

**Results:** HFA-BDP MDI ETN treatment improved all assessed clinical endpoints and corticosteroid sensitivity without any deterioration in pulmonary function. FENO and blood eosinophil number were reduced by HFA-BDP MDI ETN treatment. The visualization study suggested that ETN at expiratory flow rates of 10–30 L/min led to fine particle deposition in the middle meatus, including the sinus ostia. Co-incubation of eosinophils with BEAS-2B cells induced corticosteroid resistance.

**Conclusions:** Additional HFA-BDP MDI ETN treatment was beneficial in patients with ECRS and should be considered as a potential therapeutic option for eosinophilic airway inflammation such as ECRS with asthma. (UMIN-CTR: R000019325) (http://www.umin.ac.jp/ctr/index.htm).

## Introduction

Eosinophilic chronic rhinosinusitis (ECRS) characterized by ethmoid-predominant sinusitis with eosinophilic inflammation is a refractory airway disease categorized as a subgroup of chronic rhinosinusitis with nasal polyps (1, 2). With an increasing incidence, ECRS has been officially designated as intractable disease in Japan. Approximately half of the patients with ECRS have bronchial asthma (3), which is a risk factor of relapse after endoscopic sinus surgery (4). Furthermore, in those with severe ECRS, asthma is significantly associated with ECRS in more than 80% of cases (4, 5). Conversely, a recent report suggests that more than 50% of patients with severe asthma also had ECRS (6). Thus, ECRS should be recognized as an eosinophilic airway inflammation extending to the lower airway and treated concurrently with asthma based on the united airway concept. We recently reported on the efficacy of “airway medicine” for ECRS with asthma (7–10), where “airway medicine” refers to comprehensive care of the upper and lower airway using fine-particle inhaled corticosteroids (ICS). Fine particles travel toward the upper airway during inhalation through the mouth followed by exhalation through the nose (ETN) (7), suggesting that these fine particles could be delivered not only to the lower airway but also to the inflammatory sites in the upper airway, such as the middle meatus to which the ethmoid sinus opens. However, these reports included retrospective evaluation as a case series, and the ETN methods were inconsistent. In some cases, knowledge of the effectual flow conditions for ETN treatment improved sinusitis without any changes in treatment. Therefore, we conducted a blinded, placebo-controlled study with a consistent ETN method to confirm the beneficial effect of fine-particle ICS ETN treatment for ECRS with asthma.

## Materials and methods

### Subjects

The target population for this study was geared for patients with severe ECRS refractory to intranasal corticosteroid treatment who were referred to our department for surgical treatment. All subjects had bronchial asthma. ECRS and bronchial asthma were diagnosed according to the Japanese Epidemiological Survey of Refractory Eosinophilic Chronic Rhinosinusitis (4) and the Global Initiative for Asthma guidelines (11), respectively. Twenty-three patients were randomized in a double-blinded fashion to receive either 800 μg of HFA-BDP metered-dose inhaler (MDI) ETN (*n* = 11) or the same volume of placebo MDI ETN (*n* = 12) for 4 weeks, in addition to current therapy, which was not changed. All subjects had already stopped treatment with intranasal corticosteroids at the point of entry into the study. At the start of treatment, the method of MDI ETN was explained to all subjects as follows. Fine particles released from MDI were inhaled orally for 3 s using a valved holding chamber; the subjects then held their breath for 3 s and performed ETN for 3 s. The method of MDI ETN was based on a preliminary study in which the flow conditions of the ETN treatment was evaluated (Supplementary Material). We confirmed the subjects' technique and flow conditions under ETN treatment using a spirometer (CHESTGRAPH HI-105, Chest M.I., Tokyo, Japan) with a facemask (LiteTouch VHC Mask, Philips, Amsterdam, Netherlands). Patients covered their mouth and noses with the facemask (Supplementary Figure 1A), inhaled through the mouth, and exhaled through the nose in the manner they would when using normal ETN treatment. The effectual flow-volume curve (Supplementary Figure 1B) was obtained from each patient.

Changes from baseline in nasal polyp score (12), computed tomographic (CT) score defined by the Lund-Mackay scale (13), smell test using odor stick identification test for Japanese (OSIT-J) (14), and quality of life (QOL) score [Sino-nasal outcome test-22 (SNOT-22); The Washington University in St. Louis, Missouri (15) and Asthma Control Test (ACT) (16)] were assessed. Fractionated exhaled nitric oxide (FENO) was measured as a marker of eosinophilic airway inflammation. Response to corticosteroids was evaluated before (visit 1) and after treatment (visit 2). This study was approved by the local ethics committee of Kansai Medical University (approval number: KANIRIN1502) and registered in the University Hospital Medical Information Network in Japan (UMIN-CTR: R000019325).

### Cell preparation

Peripheral blood mononuclear cells (PBMCs) were separated by Ficoll-Paque PLUS® (GE Healthcare, Uppsala, Sweden). Eosinophils (purity > 98%) were isolated from the peripheral blood of healthy volunteers with mild eosinophilia (~4–8% of total white blood cells) by negative selection using a MACS system with Eosinophil Isolation Kit (Miltenyi Biotec, Bergisch Gladbach, Germany). The human bronchial epithelial cell line BEAS-2B was obtained from the European Collection of Authenticated Cell Culture (Salisbury, UK).

### Quantitative RT-PCR

Total RNA extraction and reverse transcription were performed using a PureLink RNA Micro kit (Invitrogen, Carlsbad, CA) and a PrimeScript RT MasterMix (Perfect Real Time; Takara Bio, Shiga, Japan). Gene transcript levels of FK506-binding protein 51 (*FKBP51*), *CXCL8*, protein phosphatase 2 catalytic subunit alpha isozyme (*PPP2CA*), and glyceraldehyde 3-phosphate dehydrogenase (*GAPDH*) were quantified by real-time PCR using a Rotor-Gene SYBR Green PCR kit (Qiagen, Hilden, Germany) on a Rotor-Gene Q HRM (Corbett Research, Cambridge, UK). Amplification primers (5′-3′) were: *FKBP51* (NM_004117), forward (F)—CAG CTG CTC ATG AAC GAG TTT G, reverse (R)—GCT TTA TTG GCC TCT TCC TTG G; *CXCL8* (NM_ 000584.3), forward (F)—ACT GAG AGT GAT TGA GAG TGG AC, reverse (R)—AAC CCT CTG CAC CCA GTT TTC; *PPP2CA* (NM_ 002715), forward (F)—CGC CAT TAC AGA GAG CCG AG, reverse (R)—TAC TTC TGG CGG CTG GTT GAG; and *GAPDH* (NM_002046), F—TTC ACC ACC ATG GAG AAG GC, R—AGG AGG CAT TGC TGA TGA TCT.

### Corticosteroid sensitivity

PBMCs were treated with dexamethasone for 45 min, followed by TNFα (10 ng/ml) stimulation overnight. The ability of dexamethasone to inhibit TNFα-induced CXCL8 release was determined in cell medium by sandwich ELISA according to the manufacturer's instructions (R&D Systems). IC_50_ of dexamethasone on CXCL8 production (Dex-IC_50_), calculated using Prism® 6.0 statistical software (GraphPad, San Diego, CA), was used as a marker for corticosteroid sensitivity. In addition, BEAS-2B cells were co-incubated with purified eosinophils overnight. After removal of eosinophils, BEAS-2B cells were treated with dexamethasone (10^−7^ M) for 45 min, followed by co-stimulation with TNFα (10 ng/ml) for 4 h. The ability of dexamethasone to enhance *FKBP51* and inhibit TNFα-induced *CXCL8* levels were evaluated by RT-PCR.

### In-cell western assay

PBMCs fixed with 4% formaldehyde for 20 min were permeabilized and blocked. Cells were incubated with primary antibodies (rabbit polyclonal antibody to phospho-glucocorticoid receptor (GR) Ser^226^; Abcam, Cambridge, UK and the mouse monoclonal antibody to GR; Santa Cruz Biotechnology, Dallas, TX) and the fluorescently-labeled secondary antibodies (IRDye 800CW goat anti-rabbit and IRDye 680RD goat anti-mouse; LI-COR Bioscience, Lincoln, NE). Ratio of fluorescence intensity of phospho-GR Ser^226^ to that of GR was analyzed by Odyssey infrared imaging system (LI-COR) according to the manufacturer's instructions.

### Cell survival

Viability of purified eosinophils was evaluated using double staining with annexin V and 7-Amino-Actinomycin D (7-AAD) (BD Pharmingen, Franklin Lakes, NJ). In some experiments, eosinophils were incubated with of IL-5 (1 ng/ml). Caspase-3 activity in purified eosinophils was assayed with a Caspase-3 assay kit (BioVision, Milpitas, CA) according to the manufacturer's instructions.

### Evaluation of deposited particles

Deposited particles were visualized in a particle deposition model using a human nasal cavity cast. Briefly, fine particles (JIS Test Powders 1, No. 11; The Association of Powder Process Industry and Engineering, Japan) with an average diameter of 2.13 μm placed in the pharyngeal side were suctioned from the external naris site under constant pressure to mimic the ETN process. In addition, in contrast to the above-mentioned method, fine particles placed in the external naris were suctioned from the pharyngeal side. Particle deposition was evaluated under direct vision.

### Statistical analysis

**Figure 1:**
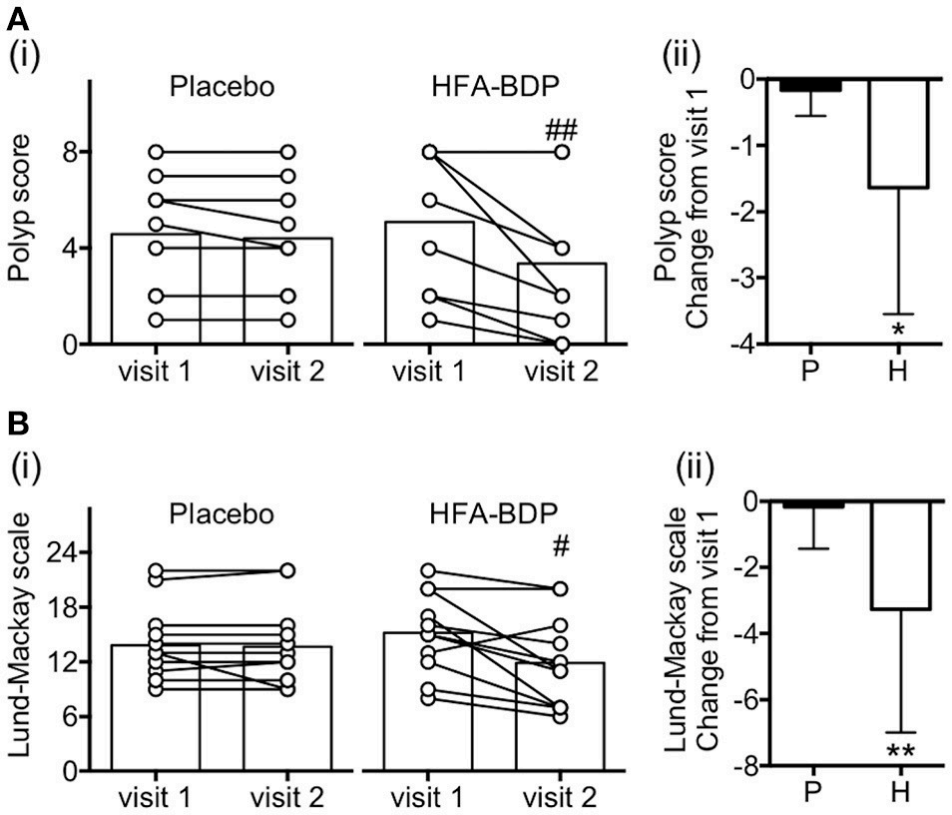
Effect of HFA-BDP MDI ETN on nasal polyp score and sinus CT findings. Nasal polyp score **(A)** and sinus CT score **(B)** were evaluated before (visit 1) and 4 weeks after treatment (visit 2). Individual values and means of 12 patients in the placebo group (P; **A** [i]) and 11 patients in the HFA-BDP group (H; **B** [i]) are shown. ^#^*P* < 0.05, ^##^*P* < 0.01 (vs. visit 1). Changes from visit 1 to visit 2 were also compared between two groups (**A** [ii] and **B** [ii]). Data represent mean ± standard deviation. ^*^P < 0.05, ^**^P < 0.01 (vs. placebo).

Comparisons of two datasets were performed using the Mann–Whitney *U*-test, Wilcoxon matched-pairs signed rank test, or Fisher's exact test as appropriate. Other data were analyzed by analysis of variance with *post-hoc* Bonferroni test adjusted for multiple comparisons. Differences were considered statistically significant if *p* was < 0.05. Descriptive statistics were expressed as means ± standard deviation (Figure 1 and Table 1) or means ± standard error of the mean (**Figure 5**).

**Table 1:** Baseline characteristics of eosinophilic chronic rhinosinusitis patients with bronchial asthma.

	**Placebo (*n* = 12)**	**HFA-BDP (*n* = 11)**
Age	50.1 ± 11.3	53.4 ± 14.3
Gender (M/F)	7/5	3/8
JESREC score	14.8 ± 1.6	15.6 ± 1.6
Severity of asthma (mild/moderate/severe)	8/2/2	5/4/2
NSAIDs intolerance	1	2
Smoking history (never/ex-smoker)	7/5	10/1
ESS history (Y/N)	4/8	2/9
Total IgE (IU/ml)	233 ± 254	280 ± 219
Eosinophils (/μL) [%]	469 ± 110 [7.6 ± 2.0]	581 ± 173 [9.5 ± 3.2]
FENO (ppb)	63.6 ± 61.5	77.6 ± 51.6
LMS (total/ethmoid)	13.8 ± 4.2/5.3 ± 1.6	15.2 ± 4.5/5.7 ± 1.5
Polyp score	4.6 ± 2.5	5.1 ± 3.1
FEV_1_ %pred.	84.0 ± 20.0	88.7 ± 17.9
FEF_25−75_ %pred.	49.1 ± 20.2	57.5 ± 24.9
FVC %pred.	99.5 ± 17.4	100.6 ± 14.4
ACT	23.0 ± 3.1	22.7 ± 3.1
SNOT-22	41.2 ± 21.8	33.7 ± 18.3
OSIT-J	3.1 ± 4.3	3.9 ± 4.5
**TREATMENT**		
ICS (μg)	150 ± 207	250 ± 314
LABA	5	4
LTRA	3	4
Anti-histamine	3	4
Theophylline	2	3
Macrolide	2	1

*ACT, Asthma Control Test; ESS, endoscopic sinus surgery; FENO, fractionated exhaled nitrogen oxide; FEV_1_, forced expiratory volume in 1 s; FEF_25−75_, forced expiratory flow between 25% and 75% of vital capacity; FVC, forced vital capacity; ICS, inhaled corticosteroid (equivalent doses of fluticasone propionate); JESREC, Japanese Epidemiological Survey of Refractory Eosinophilic Chronic Rhinosinusitis; LABA, long-acting β_2_-agonist; LAMA, long-acting muscarinic antagonist; LMS, Lund-Mackay scale; LTRA, leukotriene receptor antagonist; SNOT-22, Sino-Nasal Outcome Test-22. Values are number of subjects and mean ± standard deviation*.

## Results

### Patient characteristics

Baseline characteristics of the study patients are summarized in Table 1. There were no differences in any of the characteristics including the condition and severity of ECRS with asthma between the groups. Other medication use was similar between the two groups.

### HFA-BDP MDI ETN reduces nasal polyps and sinus opacification

After 4 weeks of treatment, the nasal polyp score was significantly improved in 8 of the 11 patients receiving HFA-BDP, compared with two of twelve patients receiving placebo (Figure 1A). Sinus CT findings evaluated by Lund-Mackay scale was also improved after HFA-BDP treatment compared with placebo (Figure 1B). Conversely, there was a significant improvement in the smell test in both groups (data not shown).

### HFA-BDP MDI ETN improves clinical markers of airway inflammation concomitant with subjective evaluation

**Figure 2:**
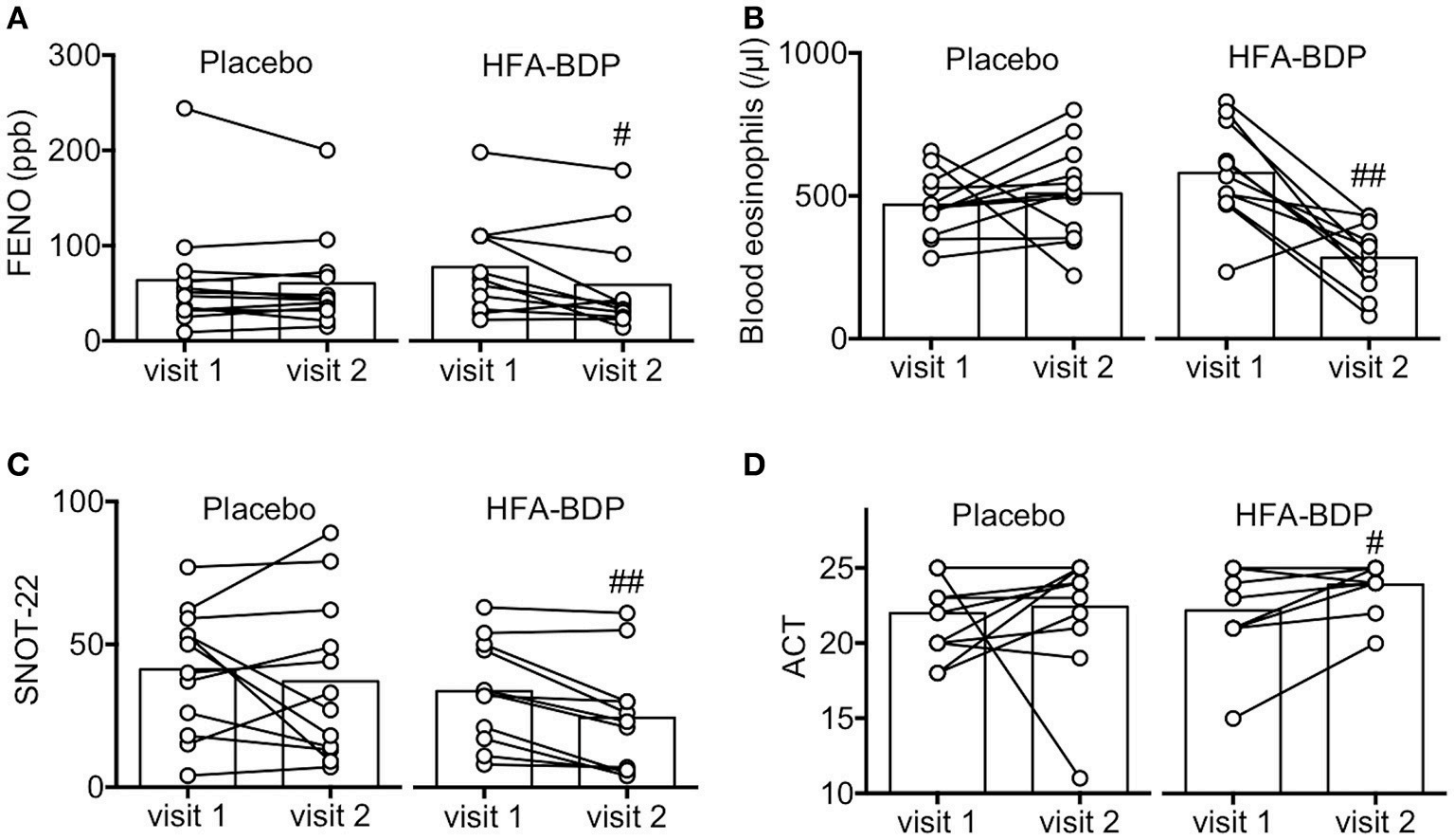
Effect of HFA-BDP MDI ETN on eosinophilic airway inflammation. Fractionated exhaled nitric oxide (FENO) **(A)** and blood eosinophil count **(B)** were measured as markers of eosinophilic inflammation before (visit 1) and 4 weeks after treatment (visit 2). Sino-nasal outcome test-22 (SNOT-22) **(C)** and asthma control test (ACT) **(D)** were also evaluated as QOL questionnaire. Individual values and means of 12 patients in the placebo group and 11 patients in the HFA-BDP group are shown. ^#^*P* < 0.05, ^##^*P* < 0.01 (vs. visit 1).

HFA-BDP MDI ETN had a beneficial effect on FENO, a clinical marker of airway eosinophilic inflammation and blood eosinophils, as well as on the findings associated with sinusitis (Figures 2A,B). In addition, reflecting the improvement in clinical findings, subjective evaluation by SNOT-22 and ACT improved significantly in the HFA-BDP group (Figures 2C,D). Regarding pulmonary function, there were no significant improvements in the indicators of airway obstruction such as %FEV1 and %FEF_25−75_ (data not shown).

### HFA-BDP MDI ETN leads to restoration of corticosteroid sensitivity

**Figure 3:**
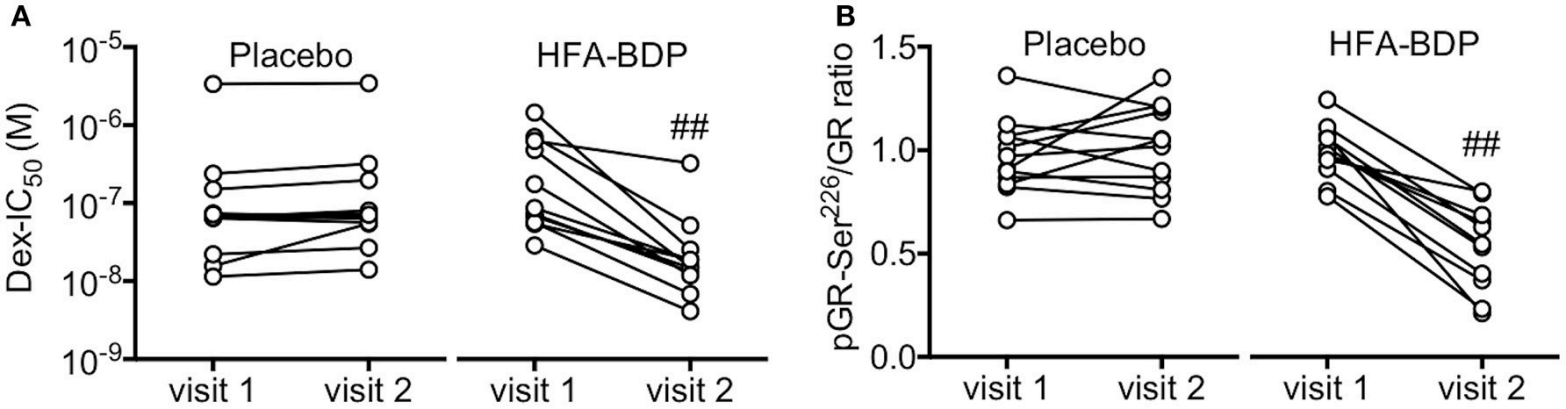
Effect of HFA-BDP MDI ETN on corticosteroid sensitivity. IC_50_ values for dexamethasone on TNFα-induced CXCL8 production (Dex-IC_50_) **(A)** and phosphorylation levels of GR-Ser^226^ **(B)** in PBMCs were measured before (visit 1) and 4 weeks after treatment (visit 2) as markers of corticosteroid sensitivity and GR inactivation, respectively. Individual values of 12 patients in the placebo group and 11 patients in the HFA-BDP group are shown. ^##^*P* < 0.01 (vs. visit 1).

The beneficial effect of HFA-BDP MDI ETN was further confirmed by a significant reduction in Dex-IC_50_ from 346.2 ± 443.6 nM before treatment to 45.9 ± 93.0 nM after treatment (Figure 3A). Supporting these findings, HFA-BDP MDI ETN reduced the increase in phosphorylation of GR at Ser^226^, a biomarker of GR inactivation (17, 18) (Figure 3B).

### Potential of fine particle deposition in paranasal sinus ostium

**Figure 4:**
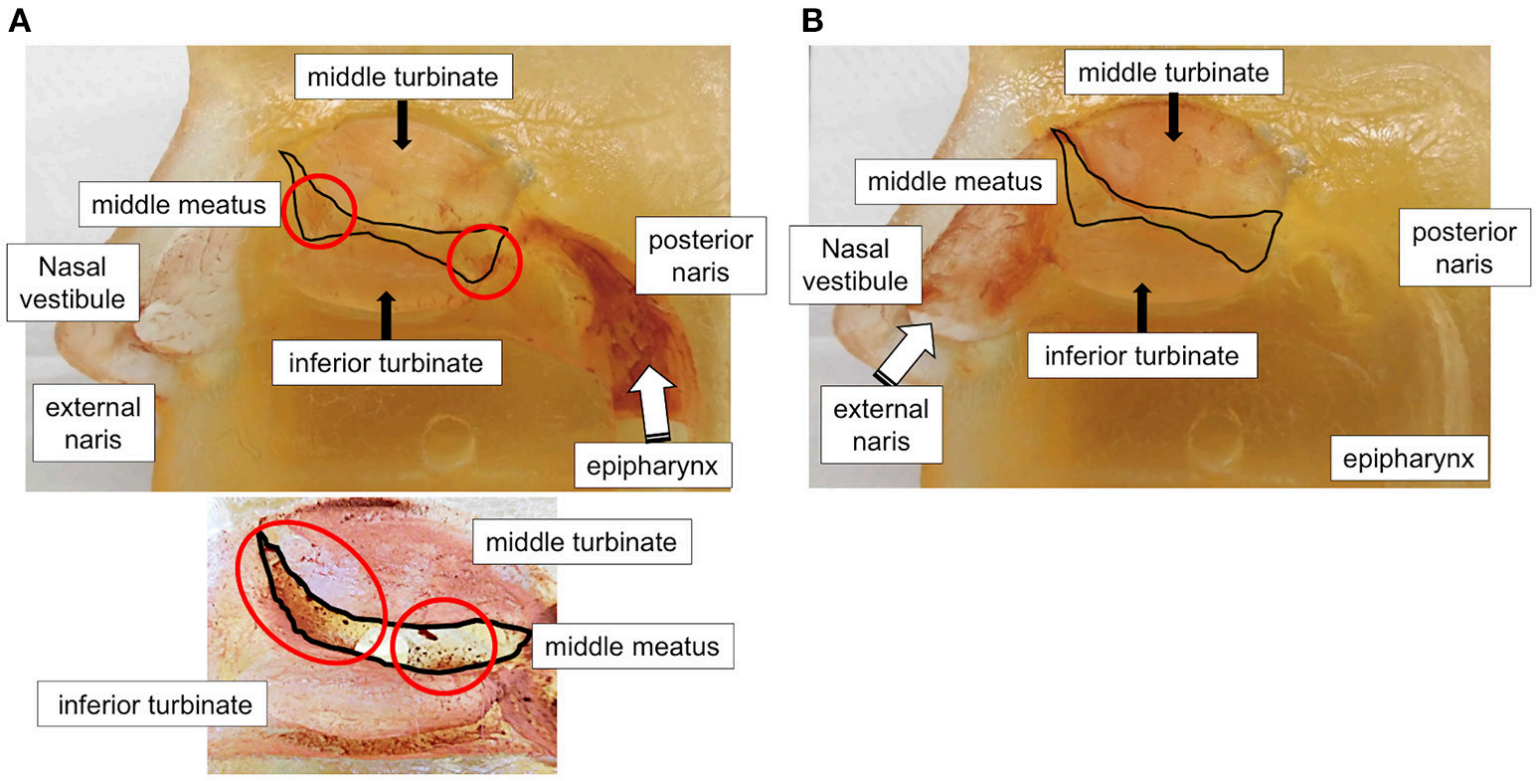
Evaluation of fine particle deposition in the nasal cavity. Visualization of fine particles in a particle deposition model using a human nasal cavity cast. Flow of fine particles from the pharynx to the external naris **(A)** and from the external naris to the pharynx **(B)** were evaluated. Lower panel in **(A)** shows the magnified view from another angle. White arrows, block circles, and brown dots and patches in red circles indicate flow direction, middle meatus area, and deposited particles, respectively.

In the particle deposition model using a human nasal cavity cast, we found that fine particles flowing from the pharynx to the external naris at flow rates of 10–30 L/min were deposited in the middle meatus where the sinus ostia are located, although the deposition was less in the epipharynx area (Figure 4A). Conversely, fine particles flowing from the external naris to the pharynx at the same flow rates were deposited mainly in the nasal vestibule and epipharynx and not in the middle meatus (Figure 4B).

### Coexistence of eosinophils reduces corticosteroid sensitivity

**Figure 5:**
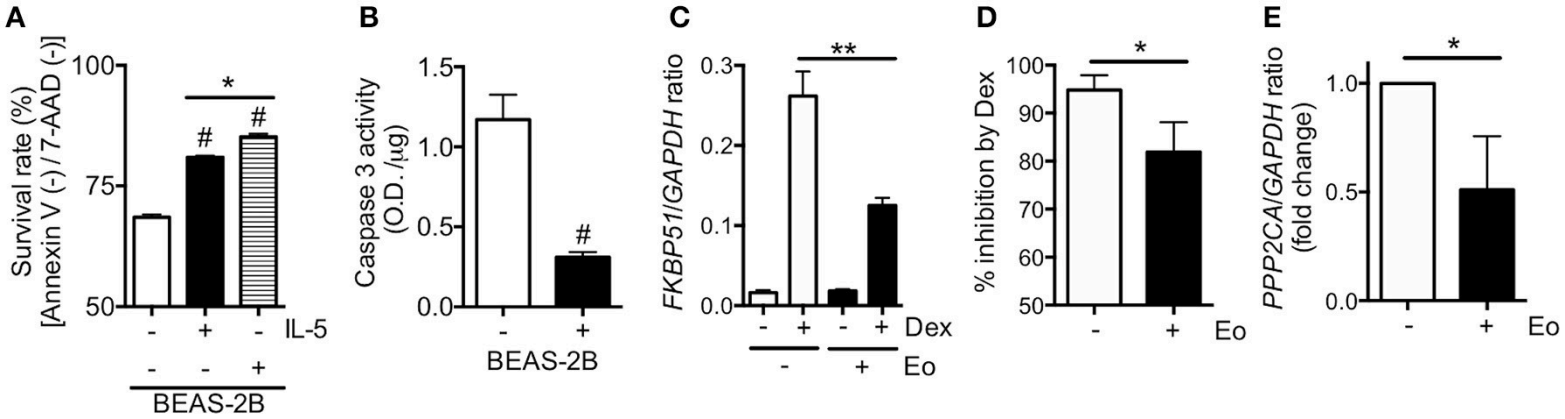
Impact of eosinophil co-incubation on corticosteroid sensitivity. Purified eosinophils were incubated with or without BEAS-2B cells overnight. Eosinophil viability **(A)** and caspase 3 activity in eosinophils **(B)** were evaluated. BEAS-2B cells were incubated with or without purified eosinophils overnight. The ability of dexamethasone (Dex, 10^−7^ M) to enhance *FKBP5* **(C)** and inhibit TNFα-induced *CXCL8* **(D)** mRNA levels were evaluated. *PPP2CA* mRNA levels **(E)** were also measured. Data represent means of three **(A,B)** or four **(C–E)** experiments ± standard error of the mean. ^#^*P* < 0.05 (vs. non-treatment control), ^*^P < 0.05, ^**^P < 0.01 (as shown between two groups).

Survival of eosinophil was prolonged when the cells were co-incubated with BEAS-2B cells, with a reduction in caspase 3 activity (Figures 5A,B). More importantly, co-existence of eosinophils significantly reduced the ability of dexamethasone to enhance *FKBP51* mRNA expression and inhibit TNFα-induced *CXCL8* mRNA expressions in BEAS-2B cells (Figures 5C,D). In line with the observed corticosteroid insensitivity, coexistence of eosinophils also reduced the mRNA expression of *PPP2CA*, a serine/threonine phosphatase PP2A catalytic subunit that plays an important role in the regulation of its complexes and activity (Figure 5E) (19).

## Discussion

The current study revealed that additional HFA-BDP MDI ETN treatment was beneficial in ECRS patients with bronchial asthma. A potential explanation is that fine-particle ICSs could be delivered by ETN not only to the lower airway but also to the upper airway sites where inflammation exists, such as the middle meatus to which the ethmoid sinus opens, supporting our findings in earlier studies (7, 9).

Although the efficacy of intranasal corticosteroids for ECRS patients was previously indicated (20, 21), their effect has been considered as incomplete and transient (22). We previously attempted to simulate fine-particles deposition using computed fluid dynamics (CFD) analysis in a three-dimensional anatomically accurate and subject-specific model reconstructed from CT data (9, 23): CFD analysis with flow rates of 30 L/min from the pharynx to the external nares revealed that fine particles (1 μm) were deposited in the nasal cavity and reached the ethmoid sinus area to a certain extent, with ~10% of the fine particles deposited in the upper airway, even in the model without endoscopic sinus surgery (ESS) (Supplementary Table 2 and Supplementary Video 1). Further, we confirmed that the fine particles released by HFA-BDP MDI at least partially (30–50%) flowed out through the external nares (Supplementary Figure 2 and Supplementary Video 2) and the possibility that fine particles might be deposited in the nasal cavity at flow rates between 15 and 30 L/min. Conversely, CFD analysis with flow from the external nares to the pharynx showed only 3% deposition on the ethmoid sinus area. This finding is supported by a previous report by Hyo et al. who reported that only 3% of the small particles in the μm range reached the paranasal sinuses (24) and by our particle deposition model with a human nasal cavity cast in the current study (Figure 4). This may account for clinical effect of fine-particle ICS ETN for severe sinusitis which is refractory to intranasal corticosteroid treatment (7, 9).

After a 4-week double-blinded period, 2 of the 12 patients in the placebo group dropped out of this study. One patient had an exacerbation of asthma, and the other patient quit follow-up at our hospital due to deterioration of ECRS. The remaining 21 patients received HFA-BDP MDI ETN treatment after the end of the double-blinded period and were followed for at least 1 year. Although all patients were referred to our department for surgical treatment, HFA-BDP MDI ETN treatment provided prolonged, good control in 10 of the 21 patients with no need for surgery. Thus, fine-particle ICS ETN treatment provided long-term benefit in severe ECRS patients with asthma. A recent large-scale study in Japan reported that ~60% of patients with severe ECRS experienced recurrence within 3 years after ESS (4). However, at our department, comprehensive care with a fine-particle ICS MDI ETN treatment reduced the recurrence rate up to 30% (data not shown).

Individuals with refractory eosinophilic airway inflammation might exhibit corticosteroid resistance, as indicated in a previous report (25). We also found that the response to corticosteroids was reduced in ECRS patients with asthma (9). Further, as shown in Figure 5, co-incubation of eosinophils with bronchial epithelial cells prolongs eosinophil survival and reduces corticosteroid sensitivity, concomitant with PP2A impairment. PP2A can regulate corticosteroid response by dephosphorylation of GR at Ser^226^ (17). Taken together, these findings suggest that inhibition of eosinophilic airway inflammation by HFA-BDP MDI ETN treatment could reduce phosphorylation levels of GR at Ser^226^, resulting in the restoration of corticosteroid sensitivity (Figure 3B). Conversely, we should consider that the effects of ETN treatment with fine-particle ICS alone, including HFA-BDP, depend on the severity of disease. For some patients with severe ECRS and asthma, ICS/LABAs are required to restore corticosteroid sensitivity (26, 9).

The current study evaluated the additional effect of HFA-BDP MDI ETN treatment which might be a potential therapeutic option for eosinophilic airway inflammation such as ECRS with asthma. Although this randomized, placebo-controlled study was conducted in a double-blind fashion, the single-center design with a small sample size is a major limitation. A multicenter clinical trial with large sample size is recommended to confirm the utility of this novel option for airway medicine based on the concept of united airway.

## Ethics statement

This study was carried out in accordance with the recommendations of Ministry of Health Labour and Welfare. The protocol was approved by the medical ethics committee of Kansai Medical University. All subjects gave written informed consent in accordance with the Declaration of Helsinki.

## Author contributions

YK, TY, MA, HY, and KT were involved in the conception and design of this study. YK and MA enrolled patients. YK, HT, TY, and AK conceived the experiments. YK, HT, TY, and AK were involved in analysis and interpretation of the data. YK, HI, HY, and KT were involved in drafting the manuscript for important intellectual content. All authors contributed to revisions and approved the final draft.

### Conflict of interest statement

The authors declare that the research was conducted in the absence of any commercial or financial relationships that could be construed as a potential conflict of interest.

## Abbreviations

ECRS: eosinophilic chronic rhinosinusitis
ESS: endoscopic sinus surgery
ETN: exhalation through the nose
ICS: inhaled corticosteroid.
