# Supplementary material for: HFA-BDP Metered-Dose Inhaler Exhaled Through the Nose Improves Eosinophilic Chronic Rhinosinusitis With Bronchial Asthma: A Blinded, Placebo-Controlled Study
Source: Front Immunol. 2018 Sep 25;9:2192. doi: 10.3389/fimmu.2018.02192 (PMC6178134; doi:10.3389/fimmu.2018.02192)
Supplement: Supplementary file 3 [file Data_Sheet_1.doc]

Supplementary Material

HFA-BDP metered-dose inhaler exhaled through the nose improves eosinophilic chronic rhinosinusitis with bronchial asthma

**Yoshiki Kobayashi, Hirotaka Yasuba, Mikiya Asako, Takahisa Yamamoto, Hiroshi Takano, Koichi Tomoda, Akira Kanda, Hiroshi Iwai**

*** Correspondence:** Yoshiki Kobayashi: kobayosh@hirakata.kmu.ac.jp

# Supplementary Material

**Supplementary Methods**

***Subjects***

To investigate the association between the efficacy of treatment with fine-particle inhaled corticosteroid (ICS) exhalation through the nose (ETN) and flow conditions under ETN treatment, 70 eosinophilic chronic rhinosinusitis (ECRS) subjects with bronchial asthma were recruited. All subjects had additionally undergone fine-particle ICS (400 or 800 μg HFA-134a-beclomethasone dipropionate [HFA-BDP]) ETN treatment for two months without any changes in other treatments. ECRS and bronchial asthma were diagnosed according to the Japanese Epidemiological Survey of Refractory Eosinophilic Chronic Rhinosinusitis Study (Tokunaga et al., 2015) and the Global Initiative for Asthma guidelines, respectively. At the beginning of treatment, the delivery method of fine-particle ICS ETN was explained to all subjects. Specifically, fine-particle ICS was orally inhaled for three seconds using a valved holding chamber; subjects then held their breath for three seconds and performed ETN for three seconds.

We retrospectively confirmed the efficacy of fine-particle ICS ETN treatment as defined by improvement of nasal symptoms (nasal congestion and anosmia) based on subjective feeling, sinus computed tomography (CT) score evaluated using the Lund-Mackay scale (Lund and Mackay, 1993), and nasal polyp score (Meltzer et al., 2006) within two months after the commencement of treatment. Briefly, subjects who fulfilled the following requirements were assigned to the responder group: improvement of (i) nasal symptoms such as anosmia, nasal congestion, and viscous rhinorrhea and (ii) sinus CT score and/or nasal polyp score. Baseline subject characteristics are summarized in Supplementary Table 1. This study was approved by the local ethics committee of Kansai Medical University (approval number: 2017035).

***Flow conditions***

We examined flow conditions under ETN treatment using a spirometer (CHESTGRAPH HI-105, Chest M.I., Tokyo, Japan) with a facemask (LiteTouch VHC Mask, Philips, Amsterdam, Netherlands). Patients covered their mouth and noses with the facemask (Supplementary Figure 1A), inhaled through the mouth, and exhaled through the nose in the manner they would when using normal ETN treatment. The flow-volume curve obtained from each patient was analyzed.

***Estimation of deposited particles***

Computational fluid dynamics (CFD) analysis was performed using a three-dimensional anatomically accurate and subject-specific model reconstructed from CT data of three subjects with endoscopic sinus surgery (ESS) and three subjects without ESS, as previously reported (Kobayashi et al., 2017; Yamamoto et al., 2017). Briefly, the surface of the nasal cavity of the model was replaced with >2 million discrete volume cells using CFD-meshing software package (ANSYS ICEM CFD Release 15.0 Manual, ANSYS Japan Inc., Tokyo, Japan) and an Euler-Lagrange particle transport model (ANSYS CFX-Solver Theory Guide Release 15.0, ANSYS Japan Inc.) with steady-state turbulent flow (expiratory flow rate, 30 L/min; particle size, 1 μm) was applied. The fine particles reaching the cells were defined as deposited particles.

***Flow image of fine particles***

The flow image of HFA-BDP MDI ETN was visualized using the Fine Particle Visualization System (Shin Nippon Air Technologies Co., Ltd., Tokyo, Japan). Briefly, the fine particles escaping through the nose after mouth inhalation followed by nasal exhalation were exposed to a laser light sheet source, “Parallel Eye H” and were detected using a professional ultrahigh-sensitive video camera.

***Fine particle count***

Using the light scattering method ‘L-Wind’ (Shin Nippon Air Technologies), the number of fine particles (size range; 0.5–5 μm) escaping through the nose after mouth inhalation followed by nasal exhalation was counted. Fine particles flowing through a lower air turbulence duct and exposed to a laser light sheet source were detected by a professional ultra-high sensitivity video camera and analyzed.

**Supplementary Results**

***Effectual flow pattern observed in patients with beneficial effects under ETN treatment***

Within two months of the commencement of additional fine-particle ETN therapy, nasal symptoms were reduced or disappeared concomitant with improvement of sinus CT score and/or nasal polyp score in 44 of the 70 patients. These patients had similar flow-volume curves as shown in Supplementary Figure 1B. Compared with the non-responders, responders exhibited relatively constant (narrow range) mid-expiratory flow rates (defined as expiratory flow between 25% and 75% of vital capacity of 31.8 ± 9.5 L/min vs. 39.1 ± 30.0 L/min in the non-responders), with higher expiratory flow volumes of 1.7 ± 0.7 L vs. 1.3 ± 0.89L in the non-responders (Supplementary Figure 1C). Flow patterns such as a higher mid-expiratory flow rate or a lower expiratory flow volume observed in the non-responders suggested that a mild expiratory flow rate with an adequate expiratory flow volume (light gray background in Supplementary Figure 1C) might provide benefit under ETN treatment.

Additional ETN treatment also led to better asthma control, supported by asthma control test scores of ≥20 points (Jia et al., 2013), with improvement in forced expiratory volume in 1 second (FEV1) and forced vital capacity (FVC) in both groups (data not shown). Furthermore, reduction in peripheral blood eosinophils from 444 ± 322 to 348 ± 296/μl and fractionated exhaled nitric oxide (FENO) from 59.8 ± 55.7 to 35.0 ± 28.9 ppb were observed only in the responders.

***Potential of fine particle deposition in the paranasal sinus ostium area***

CFD analysis with flow rates of 30 L/min from the pharynx to the external nares revealed that fine particles (1μm) were deposited in nasal cavity and reached the ethmoid sinus area to a certain extent, with approximately 10% of the fine particles deposited in the upper airway, even in the model without ESS (9.3 ± 0.6% in subjects without ESS and 27.9 ± 13.4 in subjects with ESS) (Supplementary Table 2 and Video 1).

To further assess a potential fine particle deposition in the nasal cavity, fine particles escaping through the nose during the ETN process were visualized and counted in healthy volunteers. Based on the effectual flow pattern indicated in Supplementary Figure 1, subjects orally inhaled fine particles at a peak inspiratory flow rate of about 60 L/min followed by ETN with variable mid-expiratory flow rates and an expiratory volume of about 1.7 L. The flow images using the fine particle visualization system revealed that the fine particles released by HFA-BDP MDI at least partially flowed out through the external nares (Supplementary Video 2). The percentage of fine particles (range; 0.5–5 μm) escaping through the nose was lower with mid-expiratory flow rates of 15 and 30 L/min, compared with mid-expiratory flow rates of greater than 60 L/min (Supplementary Figure 2), suggesting that fine particles might be deposited in the nasal cavity at mid-expiratory flow rates of 15–30 L/min.

**Supplementary References**

**Supplementary** **Figure and Video legends**

**Supplementary Figure 1.** Analysis of flow-volume pattern under ETN treatment. Flow-volume curve was analyzed using a spirometer with a facemask while the patients inhaled orally, followed by exhalation through the nose. (A) Facemask connected to spirometer. (B) Typical pattern of a flow volume curve of responders. (C) Comparison between mid-expiratory flow rate and expiratory flow volume. Individual values (open circle, responder; filled circle, non-responder) and mean ± standard deviation are shown.

**Supplementary Figure 2.** Estimation of fine particle deposition in the nasal cavity**.** Fine particle counts released from the nose after inhalation by mouth and exhalation through the nose. Data are expressed as returned particles defined by the ratio of particle counts before inhalation. Values represent means of three experiments ± standard error of the mean; ^#^ *P* < 0.01, ANOVA with *post hoc* Tukey’s multiple comparisons test (vs. 15 or 30 L/min). MEFR; mid-expiratory flow rate.

**Video 1.** Full movie of the fine particles escaping through the nose during the HFA-BDP MDI ETN process using the Fine Particle Visualization System.

**Video 2.** Trajectories of particle transport during ETN.

**Supplementary Table 1. Baseline characteristics of eosinophilic chronic rhinosinusitis patients with bronchial asthma**

|  | responder (n =44) | non-responder (n = 26) |
| --- | --- | --- |
| Age | 57.6 ± 11.3 | 57.1 ± 11.9 |
| Gender (M/F) | 28/16 | 13/13 |
| Severity of asthma  (mild/moderate/severe) | 23/10/11 | 10/6/10 |
| JESREC score* | 15.3 ± 1.9 | 15.4 ± 1.9 |
| NSAIDs intolerance | 8 | 6 |
| Smoking history  (never/ex-smoker) [pack years] | 24/20 [20.1 ± 13.0] | 15/11 [19.8 ± 16.1] |
| ESS history (Y/N) | 30/14 | 14/12 |
| Total IgE (IU/ml) | 323 ± 308 | 523 ± 506 |
| Eosinophils (/μL) [%] | 444 ± 322 [7.3 ± 4.8] | 381 ± 253 [6.0 ± 3.6] |
| FEV_1_ %pred. | 83.6 ± 18.0 | 81.0 ± 18.3 |
| FEF_25-75_ %pred. | 56.0 ± 25.1 | 51.1 ± 20.1 |
| FVC %pred. | 96.0 ± 13.7 | 93.7 ± 13.5 |
| ACT | 23.0 ± 3.1 | 22.7 ± 3.1 |
| LMS (total/ethmoid) | 12.2 ± 5.0/4.7 ± 1.6 | 11.6 ± 3.6/4.6 ± 1.0 |
| Polyp score | 3.3 ± 1.3 | 2.7 ± 1.1 |
| FENO (ppb) | 59.8 ± 55.7 | 44.4 ± 39.3 |
| Treatment | | |
| HFA-BDP (800 μg/400 μg) | 17/24 | 13/13 |
| Total ICS (μg) | 952 ± 399 | 856 ± 417 |
| LABA | 18 | 15 |
| LTRA | 17 | 11 |
| Anti-histamine | 5 | 4 |
| INS | 8 | 5 |
| LAMA | 2 | 2 |
| Theophylline | 2 | 1 |
| Macrolide | 4 | 3 |

ACT = asthma control test; ESS = endoscopic sinus surgery; FENO = fractionated exhaled nitrogen oxide; FEV_1_ = forced expiratory volume in 1 s; FEF_25-75_ = forced expiratory flow between 25% and 75% of vital capacity; FVC = forced vital capacity; HFA-BDP = HFA-134a-beclomethasone dipropionate; ICS = inhaled corticosteroid (equivalent doses of fluticasone propionate); INS = inhaled nasal steroid; LABA = long-acting β_2_-agonist; LAMA = long-acting muscarinic antagonist; LMS = Lund-Mackay scale; LTRA = leukotriene receptor antagonist. *JESREC score at the initial visit. Data are number of subjects and mean ± standard deviation.

**Supplementary Table 2.** Fine particle deposition ratio in nasal-pharynx area

|  | Subjects with ESS (n=3) | Subjects without ESS (n=3) |
| --- | --- | --- |
| Nasal-pharynx area  (% of total flow particles) | 22.4 ± 9.3 | 15.5 ± 4.4 |
| Ethmoid sinus area  (% of total deposition in nasal-pharynx area) | 27.9 ± 13.4 | 9.3 ± 0.6 |

ESS: endoscopic sinus surgery. Data are mean ± standard deviation.
