# Supplementary figures and images for: HFA-BDP Metered-Dose Inhaler Exhaled Through the Nose Improves Eosinophilic Chronic Rhinosinusitis With Bronchial Asthma: A Blinded, Placebo-Controlled Study
Source: Front Immunol. 2018 Sep 25;9:2192. doi: 10.3389/fimmu.2018.02192 (PMC6178134; doi:10.3389/fimmu.2018.02192)

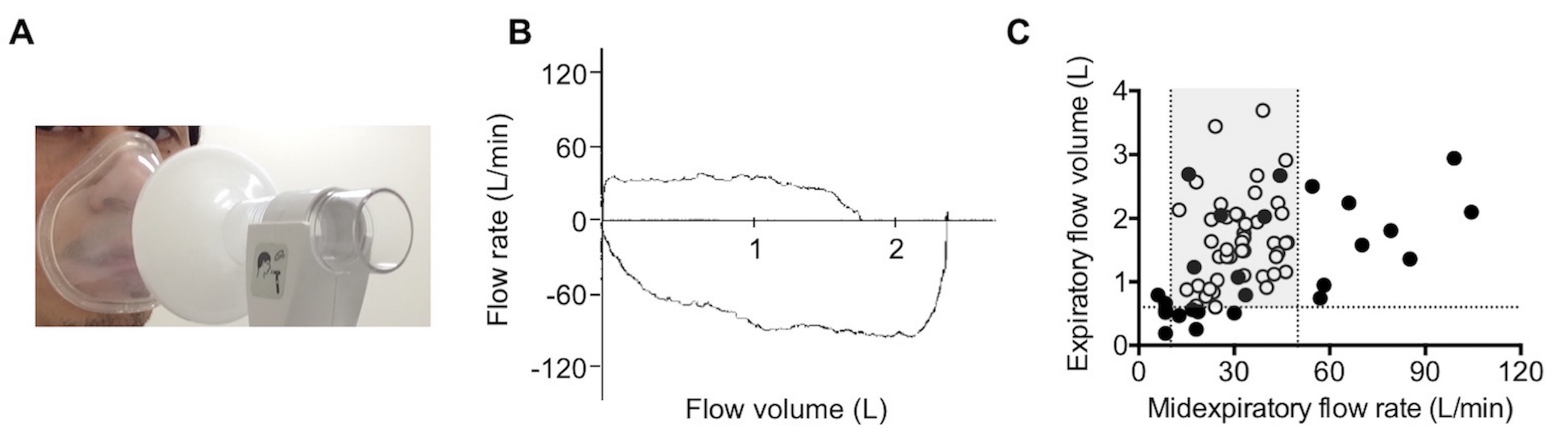

Supplement: Supplementary file 4 [file Image_1.TIFF]

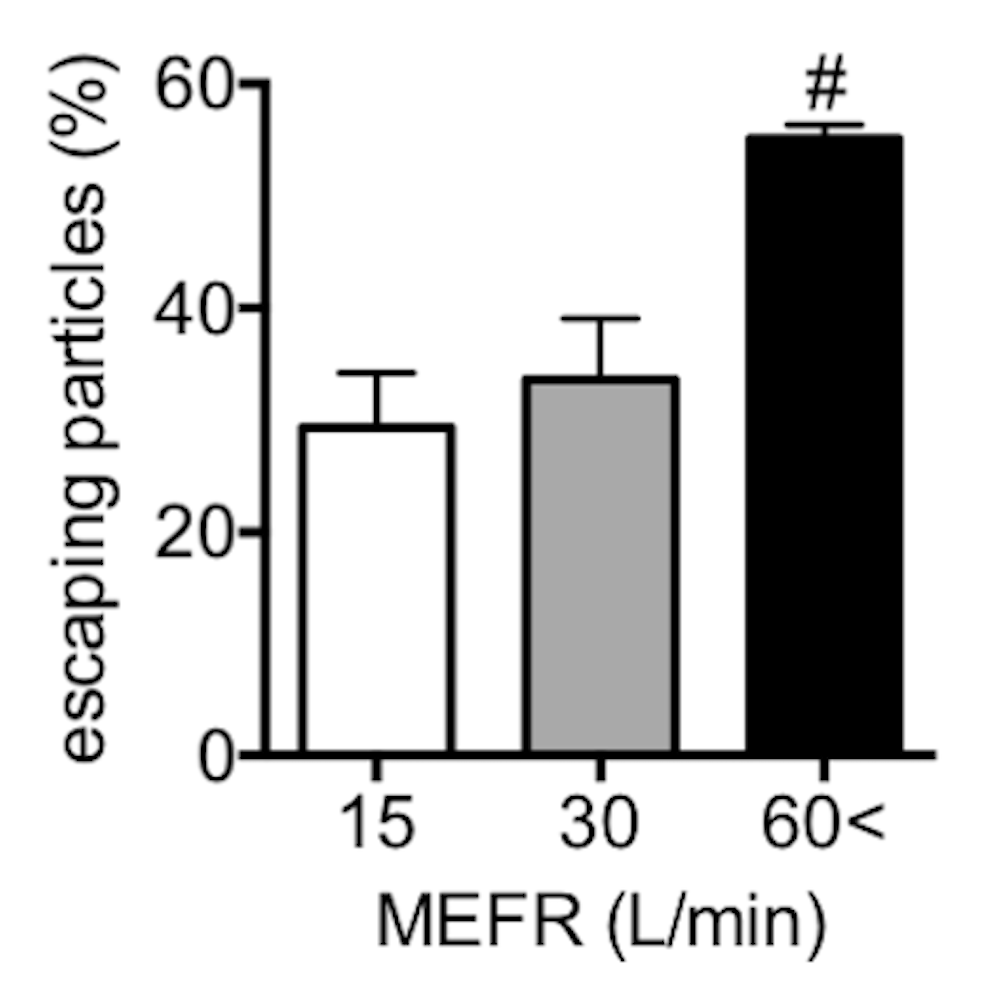

Supplement: Supplementary file 5 [file Image_2.TIFF]
